# Advances in treatment and repair technologies for exercise-induced skeletal muscle injuries

**DOI:** 10.3389/fspor.2025.1742422

**Published:** 2026-01-15

**Authors:** Cunfeng Li, Guohua Li, Haiyang Zhao, Yue Feng, Xingyu An, Xiangying Li

**Affiliations:** 1 Department of Rehabilitation Medicine, Shaoxing Hospital of Traditional Chinese Medicine, Shaoxing, China; 2 Department of Rehabilitation Medicine, The Second Affiliated Hospital of Liaoning University of Traditional Chinese Medicine, Shenyang, China; 3 Department of Endocrinology, The Fifth People’s Hospital of Shenyang City, Shenyang, China

**Keywords:** exercise-induced analgesia, inflammation, musculoskeletal pain, non-pharmacological therapy, skeletal muscle injuries, tissue engineering

## Abstract

Skeletal muscle injuries, whether acute or chronic, are a major clinical challenge due to their high incidence, persistent pain, and risk of functional impairment. While pharmacological interventions like NSAIDs and opioids remain mainstays for pain management, their prolonged use is limited by adverse effects and potential interference with muscle regeneration. Emerging evidence underscores the importance of balanced inflammatory responses in tissue repair, highlighting the need for alternative strategies. Manual therapy and exercise therapy modulate nociceptive signaling through biomechanical, biochemical, and neurocognitive mechanisms, including inhibition of central sensitization and activation of descending analgesic pathways, while advanced tissue engineering approaches (3D bioprinting, exosome therapy, and genetic engineering) directly target inflammation, enhance vascular and neuromuscular regeneration, and restore structural integrity of injured muscle. This review synthesizes current knowledge on the mechanisms underlying acute and chronic muscle injury–associated pain, emphasizing the roles of peripheral sensitization, neuroinflammation, and maladaptive central plasticity, and further delineates how specific non-pharmacological interventions are mechanistically tailored to counteract these processes.

## 1 Introduction

Skeletal muscle, comprising 40%–45% of total body mass, underpins voluntary movement, joint stability, posture, and systemic homeostasis, including respiration, thermoregulation, and metabolism (1). Despite its functional versatility, skeletal muscle is highly prone to acute damage from trauma, tumors, genetic abnormalities, or metabolic dysfunction. Such injuries often involve fiber rupture, pain, edema, hemorrhage, and impaired function (2). Chronic muscle disorders—either unresolved acute insults or primary conditions such as fibromyalgia and myofascial pain syndrome—exacerbate functional decline (3). Pain is a central determinant of recovery outcomes across both acute and chronic injuries, necessitating optimized strategies to prevent its chronification.

Clinically, pain management relies on pharmacologic and non-pharmacologic modalities. Given the incomplete understanding of peripheral and central pain pathways post-injury, pharmacotherapies such as nonsteroidal anti-inflammatory drugs (NSAIDs) and opioids are primarily symptomatic. However, long-term opioid use carries risks including dependence, gastrointestinal dysfunction, coagulopathy, and respiratory depression (4). In contrast, non-pharmacologic approaches—manual therapy (5), exercise-based rehabilitation (6), and acupuncture (7)—have gained prominence for their efficacy, safety, and ability to promote functional recovery. These interventions modulate pain through biomechanical and neurocognitive mechanisms while preserving tissue regeneration, thus representing core components of contemporary rehabilitation. This review integrates emerging clinical and mechanistic evidence to delineate the pathophysiology of muscle injury–associated pain and evaluates non-drug interventions aimed at interrupting the transition from acute to chronic pain. Elucidating these mechanisms and risk factors is critical for advancing targeted, multimodal pain management strategies in skeletal muscle rehabilitation.

## 2 Mechanisms of skeletal muscle injury

### 2.1 Risk factors for acute and chronic skeletal muscle injury

Susceptibility to both acute and chronic skeletal muscle injuries is closely linked to muscle fiber composition and surrounding tissue architecture. A hallmark of such injuries is the decline in contractile force relative to mechanical demands during activity, with key determinants including muscle activation, contraction mode, and fiber-type composition. Inadequate activation—regulated by *α*-motor neurons—where insufficient warm-up, inactivity, or poor neuromuscular control reduces motor unit recruitment and load adaptability (8); Contraction mode—eccentric contractions generate greater tension than concentric and commonly occur during deceleration, raising injury risk under fatigue or weak activation, particularly in the rotator cuff (9, 10); Fiber composition—Type II (fast-twitch, glycolytic) fibers, while power-generating, are prone to fatigue and energy depletion, reducing output and increasing injury risk under overload, exemplified in hamstring injuries (11). Together, these biomechanical and neuromuscular factors converge to heighten vulnerability to both acute and chronic muscle damage.

### 2.2 Mechanisms of acute pain following skeletal muscle injury

Pain is often the earliest and most prominent symptom of acute skeletal muscle injury, persisting throughout the pathological process. Most injuries involve partial or complete muscle fiber rupture, triggering inflammation and pain sensitization. In mild cases, vascular structures remain intact; mast cells release histamine, and fibroblasts and macrophages secrete VEGF to promote vasodilation under hypoxic stress (12). Severe injuries disrupt vasculature, activating coagulation cascades and generating anaphylatoxins, which, in turn, induce pro-inflammatory mediators such as histamine and thromboxane A2, thereby facilitating immune cell infiltration and exacerbating inflammation (13). Neutrophils infiltrate within 1 h, peak at 24–48 h, and persist up to five days, releasing prostaglandins, leukotrienes, and platelet-derived factors that sensitize nociceptors (14). Cytokines including IL-6 and TNF-α further amplify inflammation and induce secondary damage via proteases and ROS. Neutrophil apoptosis leads to monocyte recruitment and macrophage differentiation. Initially polarized to the pro-inflammatory M1 phenotype, macrophages transition around day two to anti-inflammatory M2 cells that release IL-10, supporting resolution and repair (15).

Pain during the first 72 h primarily stems from inflammatory mediators (IL-6, IL-1β, TNF-α) promoting prostaglandin and leukotriene release, which activate peripheral nociceptors. These signals travel via Aδ and C fibers to the dorsal horn, generating acute pain perception through thalamic and cortical pathways (16). Simultaneously, descending pain modulation systems involving the cortex, midbrain, and medulla are recruited. The mediodorsal (MD) thalamic nucleus plays a critical role in sustaining nociceptive signaling post-injury (17). Timely anti-inflammatory and analgesic interventions in this acute phase may attenuate peripheral nociceptive input, thus preventing central sensitization and maladaptive descending facilitation (18).

### 2.3 Mechanisms underlying chronic pain following skeletal muscle injury

Chronic myofascial pain can stem from unresolved acute injuries or primary syndromes such as myofascial pain syndrome (MPS). The “Cinderella Hypothesis” posits that sustained low-intensity contractions by small Type I motor units lead to neuromuscular overuse. These units, recruited early and disengaged last during activity, endure disproportionate metabolic stress under static conditions. Dysfunctional neuromuscular junctions release excessive acetylcholine, causing sustained depolarization, ATP depletion, and prolonged contraction. This compresses local vasculature, impairs perfusion, and promotes accumulation of algesic substances, resulting in persistent pain akin to unresolved acute injury (19, 20). Chronic peripheral nociceptive input via C fibers triggers retrograde release of substance P, activating neighboring nociceptors and perpetuating sensitization. In the spinal dorsal horn, AMPK and NMDA receptors are upregulated, while inhibitory GABAergic tone is suppressed, enhancing central nociceptive relay. Malfunction of descending inhibitory circuits further amplifies and spreads pain, often accompanied by anxiety, depression, and insomnia—hallmarks of neurocognitive dysregulation in chronic pain (21).

Beyond these neurotransmission changes, neuroimmune interactions are increasingly recognized. Activated microglia release IL-1β, TNF-α, BDNF, and ATP, sensitizing dorsal horn neurons and promoting central sensitization. Astrocytes undergo reactive transformation, altering glutamate clearance and releasing proinflammatory mediators that sustain nociceptive transmission. These glial responses are associated with synaptic remodeling—enhanced excitatory synapse density, diminished inhibition, and LTP-like plasticity in pain circuits. In parallel, cortical reorganization, especially within somatosensory and motor regions, contributes to aberrant pain processing and impaired motor control. This maladaptive plasticity sustains pain perception even after peripheral healing and underscores the need for therapies targeting central neuroimmune dysfunction.

## 3 Non-pharmacological interventions for skeletal muscle injury

NSAIDs and opioids remain first-line therapies for inflammatory pain following acute skeletal muscle injury. However, increasing evidence suggests that suppressing early inflammation can impair muscle regeneration, promote atrophy, and delay recover (22). Muscle repair is tightly regulated by a dynamic immune response, particularly the timely transition of macrophages from pro-inflammatory M1 (TNF-α, IL-1β-secreting) to reparative M2 phenotypes (secreting IL-10, TGF-β, IGF-1), which support angiogenesis, ECM remodeling, and myogenesis. This shift is orchestrated by the local cytokine milieu, mitochondrial metabolism (oxidative phosphorylation), and transcriptional regulators like STAT6 and PPARγ. NSAIDs disrupt this balance by inhibiting COX-mediated prostaglandin synthesis, affecting macrophage recruitment and phenotype switching. Opioids, via μ-opioid receptor signaling, further suppress macrophage phagocytic activity and skew polarization, impairing satellite cell activation and delaying myofiber regeneration.

Mikkelsen et al. (23) showed that indomethacin administration following eccentric contraction injury reduced satellite cell numbers and hindered regeneration in humans. Similarly, Summan et al. (24), using a skeletal muscle cryoinjury model with liposomal clodronate, demonstrated that attenuating macrophage infiltration led to persistent necrosis and adipose infiltration, highlighting the necessity of controlled inflammation for efficient repair. These studies underscore that indiscriminate or high-dose anti-inflammatory use may disrupt essential immunoregenerative processes. In contrast, non-pharmacological interventions such as acupuncture, manual therapy, and exercise offer significant analgesic effects in both acute and chronic settings. These therapies preserve or enhance myofiber contractile capacity, support tissue repair, and engage endogenous analgesic pathways involving central and peripheral modulation (18). Collectively, the data advocate for a judicious approach to pharmacologic interventions and greater integration of regenerative, non-drug modalities in muscle injury management.

### 3.1 Manual therapy

**Figure 1:**
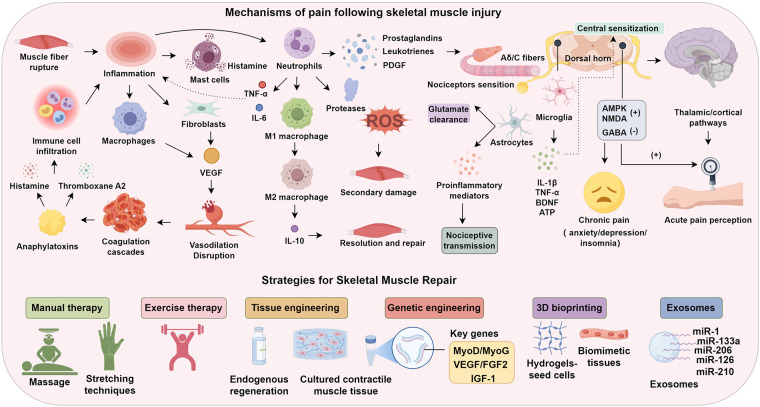
Mechanisms of pain following skeletal muscle injury.

Manual therapy, one of the oldest pain relief modalities, involves massage and stretching techniques that stimulate proprioceptors (muscle spindles, Golgi tendon organs), triggering reflexive muscle relaxation and enhancing musculoskeletal function (25). Mechanistically, it engages both neurochemical and mechanoreceptor-mediated analgesia. Activation of Aβ fibers by mechanoreceptors (including Merkel cells) leads to spinal dorsal horn modulation, inhibiting nociceptive transmission via gate-control mechanisms at lamina II. Concurrently, tactile input modulates the vagal parasympathetic axis, lowering sympathetic tone, cortisol levels, and systemic inflammation, thereby facilitating analgesia and anxiolysis. These effects span bottom-up neuromechanical circuits and top-down cognitive-emotional pathways. Smith et al. (25) demonstrated that high-intensity manual stimulation induces rapid analgesia within 20 s. In chronic pain with central sensitization, manual therapy confers durable benefits: Chen et al. (26) showed it significantly reduced spinal NMDA receptor expression in a neuropathic pain model, reversing central sensitization. Beyond somatic modulation, manual therapy influences affective processing. Repeated intervention elevates central serotonin and dopamine while reducing serum cortisol, alleviating anxiety, depression, and pain catastrophizing (27). It also activates reward-related brain regions (amygdala, nucleus accumbens, ventral tegmental area), promoting dopamine release and further reducing affective pain responses (28). These multifaceted mechanisms underscore the therapeutic relevance of manual therapy in both acute inflammatory pain and chronic maladaptive pain states, offering an integrative neuromechanistic rationale for its application across stages of muscle injury (Figure 1).

### 3.2 Exercise therapy

Aberrant biomechanical stress—whether acute from high-load forces or chronic from static postures—triggers abnormal muscle contractions, leading to skeletal muscle injury. These injuries often result in atrophy, fatty infiltration, fibrosis, strength loss, and scar formation (29), while maladaptive motor patterns further increase recurrence risk (6). Since the 1980s, clinical guidelines have evolved from “absolute rest” to encouraging minimal bed rest and sustained activity (6). Structured exercise during rehabilitation corrects biomechanical dysfunction, restores contractile control, and activates central pain modulation, resulting in exercise-induced analgesia (EIA) that alleviates inflammatory and chronic pain (30). EIA is supported by both clinical and experimental evidence. Brockett et al. (31) reported that injured muscle training mitigates strength loss and pain. In a formalin-induced myalgia model, Carmody et al. (32) found a 25% reduction in nociception after three minutes of swimming. Voluntary wheel running reduced hyperalgesia in mice compared to sedentary controls (33).

To provide a more integrated understanding of EIA mechanisms, we propose a unified model encompassing peripheral, spinal, and supraspinal components. Peripherally, exercise triggers the release of myokines—including IL-6, IL-15, and CXCL-1—that modulate nociceptive signaling, resolve inflammation, and enhance tissue repair. For instance, IL-6 exhibits a dual-phase role, transitioning from pro- to anti-inflammatory activity in bone marrow; IL-15 regulates muscle metabolism and healing; CXCL-1 promotes angiogenesis and endogenous opioid signaling (34–36). Additionally, exercise upregulates brain-derived neurotrophic factor (BDNF) in muscle, facilitating neural remodeling and reducing nociceptor excitability (37). At the spinal level, exercise downregulates excitatory glutamatergic transmission, notably via reduced NMDA receptor activation on dorsal horn neurons. Concurrently, inhibitory GABAergic and glycinergic interneurons are potentiated, diminishing central sensitization and spinal hyperexcitability (38). Supraspinally, the periaqueductal gray (PAG) and rostral ventromedial medulla (RVM) coordinate descending pain modulation. Exercise enhances the activity of serotonergic and opioidergic “OFF-cells” within the RVM, which inhibit nociceptive relay in the spinal cord, while suppressing “ON-cell” activity associated with facilitation (25). The PAG also increases enkephalin and β-endorphin production through opioid and cannabinoid receptor pathways (CB1), further reinforcing central analgesia (39, 40). Collectively, this tripartite model illustrates how exercise elicits systemic analgesia by synchronizing anti-inflammatory signaling, spinal inhibition, and descending neuromodulation, providing a mechanistic rationale for incorporating structured exercise into pain rehabilitation strategies following muscle injury.

### 3.3 Strategies for skeletal muscle repair via tissue engineering technologies

Tissue engineering for skeletal muscle repair comprises *in situ* and *in vitro* approaches (41). *In situ* methods deploy cytokine- or paracrine factor–laden scaffolds to promote endogenous regeneration, whereas *in vitro* strategies integrate seed and supportive cells with biomaterial scaffolds to construct functional muscle tissues for implantation. The latter offers enhanced spatial design, microenvironmental control, and preconditioning, improving satellite cell survival and regenerative outcomes (42, 43). Since Strohman et al. first cultured contractile muscle tissue (44), advances have included mechanical and electrical stimulation, vascular perfusion, and neuromuscular junction (NMJ) engineering. Mechanical signals modulate transcription via cytoskeletal–nuclear pathways (45), while electrical inputs enhance collagen synthesis and SC differentiation (46). Nutrient exchange remains critical. Levenberg et al. (47) established vascularized networks using myogenic, fibroblast, and endothelial co-cultures on porous biodegradable scaffolds, facilitating 3D muscle tissue construction. Pre-vascularization improves graft-host integration and accelerates functional recovery (48–50). Neuromuscular innervation is equally essential, with mature acetylcholine receptor–rich NMJs required for synchronized contraction and bioelectrical signaling. Strategies include neuronal co-culture (51) or implantation near nerve-injury zones (52) (Supplementary Table S1).

Despite progress, translational barriers persist. Mismatches in vascularization and innervation compromise graft survival, and insufficient NMJ development impairs contractile force. Limited perfusion may provoke ischemia and fibrosis. Immune rejection remains a concern, particularly in allogeneic or xenogeneic models, warranting immunomodulatory tactics or autologous sources. Moreover, current constructs rarely achieve long-term metabolic and functional integration with host tissue. Clinical translation is further hindered by high production costs, batch variability, and the complexity of fabrication processes. Addressing these limitations—through improved immunocompatibility, scalable biofabrication, and deeper mechanistic insight—will be vital to realizing the therapeutic potential of engineered skeletal muscle.

### 3.4 Application of genetic engineering in tissue-engineered skeletal muscle repair

The integration of genetic and tissue engineering offers a major advance in skeletal muscle regeneration by enabling the generation of therapeutic, gene-modified cells that promote myogenesis and vascularization. Several key genes are pivotal in these processes. MyoD and MyoG are crucial transcription factors for myogenesis, driving muscle precursor cell differentiation and the formation of new muscle fibers. Additionally, the vascularization of regenerating muscle tissue is vital for adequate nutrient supply and functional recovery. VEGF and FGF2 are well-known for their roles in promoting angiogenesis, ensuring the delivery of oxygen and nutrients to regenerating muscle tissue. IGF-1, a potent anabolic factor, has been shown to enhance muscle hypertrophy by stimulating protein synthesis and inhibiting protein degradation in muscle cells. These genes can be delivered through viral or non-viral vectors, providing a more effective means for sustained growth factor production via endogenous synthesis, thereby improving tissue regeneration and repair (53). *In vivo* methods, which embed gene vectors in scaffolds for implantation into muscle defects (54), and *ex vivo* methods, where patient-derived cells are transfected and re-implanted, are two primary strategies for gene delivery (55). Both approaches have shown promise in promoting myogenesis and vascularization, enhancing muscle repair.

### 3.5 Application of 3D bioprinting in tissue-engineered skeletal muscle repair

Over the past decade, 3D bioprinting has rapidly evolved into a powerful tool in tissue engineering, offering precise spatiotemporal control over the three fundamental elements of tissue engineering: cells, biomaterials, and bioactive molecules. Originating from conventional 3D printing technology, 3D bioprinting enables the fabrication of physiologically relevant, complex biomimetic tissues by layer-by-layer deposition of bioinks composed of biological materials, seed cells, and growth factors. Common bioprinting modalities include inkjet, microextrusion, and laser-assisted methods (56). In skeletal muscle tissue engineering, 3D bioprinting permits the precise integration of hydrogels or decellularized matrices with seed cells, faithfully recapitulating the native architecture of muscle tissue. For instance, Choi et al. (57) utilized 3D bioprinting to embed C2C12 myoblasts into decellularized porcine skeletal muscle matrix, resulting in biomimetic constructs with high myotube maturity. Subsequent studies confirmed that compared with conventional *in vitro* techniques, 3D bioprinting provides a more physiologically relevant microenvironment, promoting seed cell proliferation and myogenic differentiation (58), and facilitating enhanced integration with host tissue upon transplantation (59). Current efforts focus on optimizing bioink formulations with improved biocompatibility and mechanical integrity to further enhance the biological performance of engineered skeletal muscle. By recreating structural and biochemical features of native muscle, including vascularization and neuromuscular innervation, bioprinted tissues mitigate nociceptive input from poorly regenerated sites and reduce chronic pain susceptibility—highlighting the mechanistic justification for this intervention.

### 3.6 Application of exosomes in tissue-engineered skeletal muscle repair

Stem cell–derived exosomes (40–160 nm lipid bilayer vesicles) mimic many therapeutic effects of their source cells, acting as intercellular messengers that transfer proteins, lipids, and miRNAs (60). They promote skeletal muscle regeneration by modulating inflammation and apoptosis, enhancing angiogenesis, and supporting myogenic stem cell proliferation and differentiation (61). Exosomes help overcome limitations in tissue engineering such as seed cell scarcity, immune rejection, and poor scalability. Two main delivery strategies are used: direct injection (62), which is invasive and inefficient due to rapid clearance, and scaffold integration. Shi et al. (63) demonstrated that hydrogel-loaded exosomes enhanced muscle–tendon repair. The hydrogel network supports sustained exosome release, influencing nearby cells (64). However, conventional hydrogels lack sufficient strength and adhesion, underscoring the need for improved scaffold materials to advance exosome-based therapies. Importantly, the therapeutic efficacy of exosome-based interventions depends heavily on their molecular cargo, especially the composition of proteins and regulatory miRNAs they carry. Myogenic miRNAs such as miR-1, miR-133a, and miR-206 are well-documented for promoting muscle differentiation and regeneration, while angiogenic miRNAs like miR-126 and miR-210 contribute to neovascularization and tissue perfusion. The ability of exosomes to deliver these specific cargos to target sites is pivotal for their regenerative effects (65). Nevertheless, exosome heterogeneity—arising from differences in donor cell type, culture conditions, and isolation methods—poses major challenges to reproducibility and clinical standardization. Additionally, there is a lack of consensus regarding dosing metrics (exosome number vs. total protein content), which further complicates cross-study comparison and regulatory approval (66). Thus, future research should emphasize cargo profiling, batch quality control, and biomaterial compatibility to ensure therapeutic consistency and safety.

## 4 Conclusion

Skeletal muscle injury–associated pain involves dynamic peripheral and central mechanisms—acute phases driven by inflammatory mediators and chronic stages marked by neuroplastic maladaptation. While NSAIDs and opioids alleviate symptoms, they may hinder regeneration and fail to target underlying pathologies. Non-pharmacological interventions, including manual therapy and exercise rehabilitation, restore biomechanics, enhance endogenous analgesia, and address psychosocial comorbidities. However, heterogeneity in treatment response, absence of standardized protocols, and limited reproducibility constrain their translational utility. Recent tissue engineering advances—3D bioprinting, exosome-based therapies, and genetic engineering—recapitulate native microenvironments and promote myogenesis, but remain challenged by graft integration, functional longevity, and manufacturing scalability. Moreover, the diversity of injury phenotypes necessitates individualized rehabilitation frameworks tailored to patient-specific pathophysiology and recovery trajectories. Crucially, our incomplete understanding of the cellular and molecular processes driving pain chronification limits effective prevention of persistent musculoskeletal pain. To bridge these gaps, future studies should define dose–response dynamics of non-drug therapies, refine bioactive delivery systems, and employ integrative approaches aligned with injury-specific mechanisms. The convergence of mechanistic insight and translational innovation offers a promising avenue to transform skeletal muscle injury management and improve patient outcomes.
